# The Role of Probiotics in Inflammation Associated with Major Surgery: A Narrative Review

**DOI:** 10.3390/nu15061331

**Published:** 2023-03-08

**Authors:** Rafail Matzaras, Nikolaos Anagnostou, Anna Nikopoulou, Ilias Tsiakas, Eirini Christaki

**Affiliations:** 1 Infectious Diseases Unit, Department of Medicine, University General Hospital of Ioannina, University of Ioannina, St. Niarchou, 455 00 Ioannina, Greece; 2 Faculty of Medicine, University of Ioannina, 451 10 Ioannina, Greece; 3 Department of Internal Medicine, G. Papanikolaou General Hospital of Thessaloniki, 570 10 Thessaloniki, Greece

**Keywords:** microbiota, gut microbiota, inflammation, surgery, dysbiosis, probiotics, symbiotics

## Abstract

Background: Gut microbiota is well-known for its ability to maintain intestinal homeostasis. However, the disruption of this homeostasis, known as dysbiosis, leads to multiple consequences, including local and systemic inflammation. Surgery-induced inflammation is a major concern for patients, as it leads to many infectious and non-infectious complications. Objective: The purpose of this review was to explore the role of probiotics and symbiotics in surgery-induced inflammation and to determine if their use is effective in combatting inflammation and its complications Methods and Materials: A literature search was conducted, and articles published only in English, until December 2022 were included. The results are reported in the form of a narrative review. Results: The perioperative use of probiotics and/or symbiotics results in lower risk of infectious complications, including reduced rates of surgical site infections, respiratory and urinary tract infections, shorter hospital stays, and fewer days of antibiotic administration. It also contributes to reducing non-infectious complications, as it mitigates systemic and local inflammation via maintenance of the intestinal barrier, improves intestinal mobility, and is associated with lower rates of postoperative pain and anastomotic leak. Conclusions: Restoring gut microbiota after disruptions caused by surgery may accelerate local healing processes, attenuate systemic inflammation, and may thus prove beneficial to certain populations.

## 1. Introduction

Gut microbiota is a community of microorganisms, consisting of bacteria, fungi, archaea, protozoa, and viruses. All these different microorganisms live symbiotically in the intestinal tract and interact with human organisms through different pathways [1]. It is estimated that about 10^14^ bacteria inhabit the human gut and belong to approximately 3500 to 4000 species [2]. Gut microbiota is dominated by two major phyla; *Bacteroidetes* and *Firmicutes*, whereas *Actinobacteria*, *Proteobacteria*, and *Verrucomicrobia* are found considerably to a lesser extent [3]. The composition of gut microbiota begins immediately after birth, and various factors can modify its composition and function. Nevertheless, the distribution of these bacteria is highly variable among individuals, and their composition can be influenced by a multitude of factors [1]. Those factors include age, sex, delivery method, nutrition, drugs, genetics, ethnicity, race, environmental conditions, and lifestyle changes [4]. It is known that microbial composition is unique for every individual although the proportion of the major phyla inhabiting the gastrointestinal tract (GIT) can vary. In trials conducted both in humans and animals, it was shown that beneficial GIT microbiota can be disrupted by various factors, including dietary changes, antibiotic use, psychological and physical stress, radiation, altered GIT peristalsis, gastrointestinal infections, and surgery, and such dysregulation has been linked to infection and inflammation [5]. Among them, and excluding non-modifying factors, such as genetics or the normal evolution of microbiota from early to elderly life [6,7], antibiotic exposure and lifestyle along with diet have been widely associated with disruption of gut microbiota [8]. Antibiotic use, depending on the timing, duration, or spectrum of antibiotics used, could have negative regulatory effects on gut microbiota by reducing the bacterial diversity and enhancing the colonization of pathogenic and resistant bacteria [9,10]. Similarly, gut microbiota is very susceptible to changes due to alterations in diet content and quantity [11]. Many different types of diets were studied on different continents and populations, but except for the beneficial role of a high-fiber diet, there is no clear evidence that a specific diet is advantageous or harmful for gut homeostasis [12]. However, it could be implied that the adoption of a Westernized diet has negative implications on human gut microbiota [13,14]. Indeed, a Western diet, a diet characterized by high consumption of fats, processed meats, refined sugars, added conservatives, and low intakes of unprocessed food and fibers [15,16], has been linked with the occurrence of low-grade systemic inflammation due to the disruption of the gut barrier, the development of gut dysbiosis, the increase in intestinal permeability, and the leak of toxic metabolites into blood circulation [17,18].

The role of probiotics and symbiotics in modulating gut microbiota and preserving gut homeostasis has been widely studied. Their immunomodulatory properties seem to play an important role in promoting gut health through various mechanisms, such as strengthening of the gut epithelial barrier and regulation of mucus secretion or even modulation of immunoglobulins and cytokines [19,20]. Therefore, they seem to contribute to supporting the host immune system and attenuating local and systemic inflammation [21,22]. However, there is insufficient information on the role of probiotics and symbiotics in controlling gut dysbiosis and subsequent local or systematic inflammation provoked by major surgery. For this reason, our narrative review aims to thoroughly explore the research efforts which have taken place in this field (from experimental models to clinical studies and from pathophysiological pathways to interventional trials), in order to extract useful conclusions for future research and clinical practice.

## 2. Materials and Methods

Only peer-reviewed journal papers published until December 2022, written in English, encompassing clinical trials and animal studies were included. Papers were excluded if they did not fit into the conceptual framework of the study. In order to identify potentially eligible documents, a literature search through MEDLINE and Google Scholar was conducted. However, only journals indexed in PubMed were included. In addition, the reference lists of the initial papers were searched for further relevant articles. The search strategy was planned, defined, and executed by all authors. RM, NA, and AN assessed the titles, abstracts, and text of eligible publications found in their research. Any disagreements in selecting studies or extracting information were resolved by discussion with EC and other authors. Both qualitative and quantitative data were extracted. Based on their interpretation, important results were extracted, and a narrative review was synthesized, based on the PRISMA-ScR (Preferred Reporting Items for Systematic reviews and Meta-Analyses extension for Scoping Reviews) guidelines [23].

## 3. Results

### 3.1. Role of Gut Microbiota in Intestinal Homeostasis and Function

Gut microbiota imparts substantial functions, such as nutrition metabolism, xenobiotic and drug metabolism, antimicrobial protection, immunomodulation, vitamin production, and integrity of the gut epithelial barrier [2]. Specifically, gut microbiota is responsible for the fermentation of carbohydrates that have escaped the absorption from the small intestine by specific bacteria, such as *Bacteroides*, *Roseburia*, *Bifidobacterium*, *Fecalibacterium*, and *Enterobacteria*, to short chain fatty acids (SCFAs), such as butyrate, acetate, and propionic acid [2]. Butyrate can prevent the accumulation of toxic metabolites such as D-lactate. In addition, gut bacteria are responsible for the production of a variety of metabolites, such as histamine and Gamma-aminobutyric acid (GABA) [2]. As far as immunomodulation is concerned, it is now known that SCFAs, and especially butyrate, activate G-coupled receptors and is involved in the epigenetic modification of genes found in Treg cells [2]. 

Currently, there is emerging evidence supporting the role of gut microbiota in maintaining the gut epithelial barrier by inducing the expression of proteins of tight junctions between epithelial cells [2]. Gut bacteria excrete factors, such as butyrate and small proline-rich protein 2A (sprr2A), which enhance the synthesis of proteins, such as occludin, claudin, and zonula occludens in tight junctions, and help maintain the desmosomes in the epithelial villi [2,24]. Other studies have highlighted the healing role of gut microbiota by inducing reactive oxygen species (ROS) production by epithelial cells. Normally, ROS promote the proliferation and enhance the intestinal barrier function. Lactobacilli, for example, demonstrate this feature by inducing ROS regeneration in epithelial cells [25]. Additionally, gut bacteria produce specific metabolites that induce synthesis of antimicrobial proteins (AMPs) in the small intestine, such as cathelicidins, C-type lectins, and (pro)defensins, by the host’s Paneth cells [2]. Moreover, microbial elements and metabolites exert anti-inflammatory or pro-inflammatory effects by interacting with macrophages [26]. For example, upon interaction with gut microbiota, macrophages seem to play a role in the pathophysiology of irritable bowel disease (IBD), due to a switch from the M2 phenotype, with anti-inflammatory function, to M1 phenotype, which exerts pro-inflammatory functions [26]. M1 macrophages exert their function under the influence of interferon-γ (INF-γ) and lipopolysaccharide (LPS), whereas M2 macrophages are under the influence of interleukin-4 (IL-4) and interleukin-13 (IL-13) [26]. For all these reasons, it has been made clear that gut microbiota play an important role in human health, which probably justifies naming it, “the forgotten organ” [26].

### 3.2. The Role of Gut Microbiota in Intestinal and Systemic Inflammation

During the last decade, it has become widely appreciated that gut dysbiosis is linked to the pathogenesis and progression of a wide variety of inflammatory conditions, including autoimmune diseases and systemic infections. It has been described that in IBD the numbers of commensal bacteria, such as Firmicutes and *Bacteroidetes*, were reduced, whereas the numbers of *Enterobacteriaceae* were increased [26]. Moreover, it is known that Adherent-invasive *E. coli* (AIEC) is strongly associated with Crohn’s disease (CD) and Diffusely adherent *E. coli* (DAEC) has been linked to ulcerative colitis (UC) [26]. In addition, SCFA producing bacteria and genes involved in SCFAs metabolism have been described in CD [3]. Other autoimmune diseases have also been linked to gut dysbiosis, including multiple sclerosis (MS), rheumatoid arthritis (RA), and systemic lupus erythematosus (SLE) [27]. As far as microbiota synthesis is concerned, it has been discovered that *Bacteroides* species play an important role in the host’s immunity by preventing the excessive colonization of potential pathogenic bacteria. Given that fact, a reduction in Bacteroides species is linked to IgE-induced food allergies and IBD in children [28]. Therefore, a reduction of *Bacteroides* species following intestinal surgery could explain the increase of interleukin-12(IL-12) and interleukin-18 (IL-18), which are believed to be produced in response to bacterial products that have priorly initiated adaptive and innate immune responses [28]. 

Furthermore, stress is thought to be able to induce changes to the bacterial composition through the gut–brain axis, affecting gut permeability and favoring bacterial translocation through a corticotropin-mediated mechanism or by facilitating the entry of LPS in systemic circulation and subsequent inflammatory responses. Even after severe burns, the gut microbiota composition can undergo rearrangement showing a 90% reduction of *Bacteroides* and *Firmicutes* and increase of *Proteobacteria* such as *E. coli* [25]. Studies from critically ill patients in intensive care units (ICU) have shown that during sepsis, gut microbiota can undergo changes in composition characterized by an increase in *Clostridia* spp. and *Enterococcae* spp. and a reduction of SCFA-producing bacteria, such as *Faecalibacterium* spp., *Prevotella* spp., and *Blautia* spp. [29]. Additionally, metabolites, such as SCFAs, which promote tight junction maintenance and decrease epithelial gut permeability have been found in reduced amounts compared with healthy controls. That could potentially contribute to compromised gut integrity and systemic manifestations in sepsis [29]. However, the validity of these findings should be further explored, as there is limited evidence supporting this hypothesis.

### 3.3. Disruption of Gut Homeostasis in Surgery and Its Effect on Systemic Inflammation

As stated above, stress can induce alterations in the composition of gut bacteria through corticotropin-mediated pathways, which can further lead to increased gut epithelial permeability and activate systemic inflammatory responses [25]. Additionally, exposure of lumen bacteria to oxygen during colorectal surgery can cause changes to gut bacterial composition [25]. That can be explained by the fact that the majority of gut bacteria are facultative anaerobes or obligate anaerobes, balanced throughout lifetime [25]. Open bowel surgery can lead to the loss of anaerobic bacteria, such as *Bacteroides*, which are perceived as beneficial microorganisms, and the gain of facultative anaerobes, such as Enterococcus, in rat models which are considered harmful microorganisms [25]. Other studies have shown that ischemia of the gastrointestinal tract can lead to alterations in bacterial composition in rat models [25]. It has also been theorized that alterations in the gut microbiota due to surgical interventions and according to the type of bowel reconstruction, can lead to luminal leakage, which is responsible for the malabsorption and maldigestion of nutrients, especially in bariatric surgery [25]. Given that bariatric surgery (BS) is the best studied surgical procedure concerning its effect to gut microbiota synthesis, vitamin deficiencies have been described and are considered to be one of the most important and immediate imbalances observed following BS [30]. Characteristically, vitamin B12 is found mostly impaired because of reduced surface for its absorption after BS and reduced secretion of the intrinsic factor, which is thought to be the main cause for B12 deficiency postoperatively [30]. Other vitamin deficiency, such as B1, is associated with the onset of pathologies, such as Wernicke’s encephalopathy, that is observed in postoperative patients who underwent Roux-en-Y gastric bypass (RYGB) or vertical sleeve gastrectomy (VSG) surgery [30]. Moreover, chemotherapy and radiotherapy are also able to induce alterations in gut bacterial composition [25]. 

Postoperative ileus is a usual complication following abdominal surgery and has been linked with various mechanisms, such as inflammation, inhibitory neural reflexes, and neurohormonal peptides [25]. Through these pathways, the bacterial composition can influence gastrointestinal motility [25,31]. IL-12, for example, secreted by dendritic cells, can stimulate the production of IFN-γ by T-helper 1 (Th1) which leads to inducible nitric oxide synthase (iNOS) production by macrophages. The iNOS production inhibits contraction in smooth muscle cells of the bowel and leads to postoperative ileus [25,31]. In their review, Agnes et al. report that surgical site infections are one of the most frequent postoperative complications, where *E. coli*, *P. aeruginosa*, and *Enterococcus* spp. have the highest prevalence [25]. Therefore, it is suggested that gut microbiota modulation may exert beneficial effects on preventing postoperative complications following surgical procedures [25]. Another surgical complication, deep organ surgical site infection, is thought to be associated with anastomotic leakage following colorectal surgery [25]. In their review, Agnes et al., mention that gut microbiota promotes, through various pathophysiological mechanisms, anastomotic healing [25]. They also report that anastomotic leakage is associated with low bacterial diversity and particularly an abundance of *Bacteroidaceae* and *Lachnospiraceae* [25]. Finally, they conclude that an abundance of *Enterobacteriaceae* and the predominance of low bacterial diversity is correlated with an increased incidence of anastomotic leakage and therefore multiple adverse postoperative outcomes [25].

### 3.4. Role of Probiotics and Symbiotics in Surgery-Induced Inflammation

There are numerous randomized clinical trials (RCTs) and meta-analyses in the literature demonstrating the potentially positive effects of probiotics and/or symbiotics on patient outcomes after surgery [32,33,34,35,36,37]. The majority of the studies involve patients who have undergone colectomy for malignant disease of the gastrointestinal tract [32,33,34,35,36,37,38]. There are also studies in patients with hepatobiliary cancer [39] or pancreatic cancer [40], and in liver transplant patients [41,42,43]. Therefore, there is increasing evidence that the use of probiotics is potentially associated with a reduction in the incidence of infectious and non-infectious complications.

#### 3.4.1. Infectious Complications

A recently published meta-analysis showed that patients receiving probiotics and symbiotics preoperatively or pre and postoperatively had a lower risk of respiratory infections (risk ratio (RR) 0.35 [0.20–0.63]), urinary tract infections (RR 0.41 [0.19–0.87]), and surgical site infections (RR 0.70 [0.52–0.95]) than the control group [33]. Consistent with these findings are the results of another study which recorded a 37% reduction in the incidence of all infectious complications after surgery when probiotics were used, showing that administration of these regimens may be a promising preventive method in clinical practice [35]. Additionally, a meta-analysis of 34 RCTs involving 2723 patients undergoing elective abdominal surgery found that administration of probiotics or symbiotics perioperatively resulted in a significant reduction in infectious complications. This positive effect was greater with the use of symbiotics than when probiotics were administered alone. In addition, patients in the symbiotic group had a shorter hospital stay [44]. Kinross et al. found a lower incidence of postoperative sepsis and lower use of antibiotics after surgery when symbiotics were used perioperatively [45]. A study by Zeng et al. showed similar results in terms of postoperative infections, while reductions in inflammatory markers, gut dysbiosis, and non-infectious complications were also observed [46]. 

A meta-analysis of studies investigating the effect of probiotics on postoperative infections in certain surgical procedures also showed similar results. In the study by Tang et al. with patients undergoing pancreaticoduodenectomy, the administration of probiotics reduced the incidence of postoperative infections, gastric emptying time, duration of antibiotic therapy, and length of hospital stay, without affecting mortality [40]. The positive effects of probiotics on reducing postoperative infections have also been demonstrated in liver transplant patients. A meta-analysis of 4 studies with 246 participants showed that transplant patients who received probiotics developed a lower rate of postoperative infections (infection rate 7% vs. 35%) while having a shorter duration of antibiotic treatment and fewer hospital days in both the ICU and the hospital [43]. Similar results in transplant patients were also found in a recent meta-analysis [47]. 

Regarding the beneficial effect of probiotics on ventilator-associated pneumonia (VAP) in mechanically ventilated critically ill ICU patients, there are conflicting results. Zeng’s study found that probiotics can prevent VAP [48]. The results of a Cochrane meta-analysis showed that the use of probiotics reduced the incidence of VAP, but no firm conclusions can be drawn due to heterogeneity of studies regarding the dose and type of probiotics and the small number of participants [49]. However, another study showed that critically ill patients who received probiotics had a shorter stay in the ICU and the hospital but a similar incidence of VAP [50]. The beneficial effect of probiotics on lung infections may be related to the effect of gut microbiota on lung immunity, known as the gut–lung axis [51].

It also appears that probiotics can reduce surgical site infections (SSIs). A meta-analysis of 20 studies involving 1374 patients undergoing abdominal surgery showed that patients in the probiotics/symbiotics group were 37% less likely to develop SSIs than participants in the placebo or standard treatment group (Relative Risk (RR): 0.63; 95% Confidence Interval (CI): 0.41–0.98; N = 15 studies) [52]. According to Liu et al., only probiotics with multiple strains have a positive effect on the incidence of infections (Odds Ratio (OR) = 0.30, 95% CI: 0.15–0.61, *p* = 0.0009), including both SSIs (OR = 0.48, 95% CI: 0.25–0.89, *p* = 0.02) and non-SSIs (OR = 0.36, 95% CI: 0.23–0.56, *p* < 0.00001) [53]. A lower incidence of SSIs (22.7% versus 36.0%, *p* = 0.05) was also found in a post hoc analysis of critically ill multi-trauma patients taking a four-probiotic regimen [54]. 

It is thought that the main Gut–Brain–Skin mediator is the hypophysial hormone oxytocin, which is believed to be increased following probiotic supplementation [25]. It has a crucial role in wound healing and repair capacity in mice and humans, and therefore, supplementation with probiotics might prove beneficial for reducing surgical site infections and wound healing time [25]. Indeed, as shown in animal studies, probiotics can improve vascularization and epithelialization, reduce microbial load, and inhibit biofilm formation, all of which have a positive impact on wound healing [55].

#### 3.4.2. Non-Infectious Complications

##### Local Inflammation

- Permeability of Intestinal Barrier

The use of probiotics contributes to preserving intact intestinal mucosa, as shown by the expression of occludin in the epithelial cells of the intestine and the decreased ratio of lactulose to mannitol (L/M) [56,57]. In one RCT where subjects were given probiotics once daily, 6 days before, and 10 days after colectomy for colon cancer, it was found that the day before surgery and 10 days postoperatively, the L/M ratio was significantly lower in the probiotics group [58]. However, on the third day after surgery, the L/M ratio was similar between the two groups meaning that the stimulus from the operation may influence the effects of probiotics. Similar results with a reduced L/M ratio 10 days postoperatively were presented in another study [24]. Some studies collected segments of the normal colon during surgery to examine the effect of probiotics on colon epithelium. By calculating the tissue conductance using Ohm’s law (passive permeability to ions), they found that the mean transepithelial resistance was higher in patients who had taken probiotics, and by examining the transepithelial flux of horseradish peroxidase, they revealed a reduced permeation of peroxidase in the probiotics group [24,58]. These two findings are indicative of the preservative action of probiotics at the colonic epithelial barrier. Probiotics are also found to be effective in maintaining intestinal tight junctions [24]. The perioperative use of pro/symbiotics led to a preserved or even increased expression of tight junction proteins, such as Claudin-1, Junction Adhesion Molecule-1 (JAM-1), and occludin [24]. On the other hand, in two other RCTs where biliary cancer patients received pre- and postoperative symbiotics, there was no important difference in postoperative L/M ratios when compared to the control groups [59,60].

Another marker for investigating intestinal permeability is zonulin, as its increase shows an impaired gut barrier [61]. Zonulin is a human protein that resembles the Zonula occludens toxin which derives from Vibrio cholerae [62]. It is found that zonulin induces the disassociation of tight junctions resulting in increased intestinal permeability [61,62]. Zonulin levels are elevated in many conditions that involve chronic inflammation [63]. Probiotics are found to decrease zonulin concentration in the serum postoperatively and subsequently protect intestinal mucosa [58]. The same results were reported in another RCT, including patients with colorectal cancer and liver metastases who underwent surgery for their liver metastases. They found that the levels of zonulin in the plasma of patients that received probiotics perioperatively were surprisingly lower than the control group 10 days after surgery [64].

Another way to examine the prosperous effect of perioperatively administered pro-/symbiotics in the intestinal barrier is by measuring the levels of short-chain fatty acids (SCFAs), such as butyric acid, acetic acid, and propionic acid, which have been found to contribute to the normal function and protection of the intestinal barrier [65,66]. Therefore, an increase in levels of SCFAs after probiotic or symbiotic use is indicative of their protective role in intestinal permeability [67]. In four RCTs where patients received symbiotics perioperatively before major surgery (either hepatectomy or colectomy), the levels of SCFAs were significantly elevated in the symbiotic group in comparison to the control group, manifesting the beneficial role of symbiotics on the intestinal barrier during surgery [59,60,68,69,70]. Similar results were presented in two other RCTs where patients with esophageal cancer received symbiotics perioperatively, and they had increased or stable levels of SCFAs after esophagectomy in contrast to the control group [70,71]. However, in a small RCT with liver cancer patients who underwent hepatectomy, the perioperative use of symbiotics led to a decrease in SCFAs levels postoperatively instead of increasing them [72]. On the other hand, there are some studies with neutral results regarding the effect of perioperative use of probiotics or symbiotics on intestinal permeability as they have shown no significant difference or protective role [73].

Secretory Immunoglobulin A (sIgA), as a first-line defense mechanism, plays an important role in preventing or reducing local inflammation [74]. Thus, it can be assumed that IgA production or maintenance, induced by pro-/symbiotic use, could assist in reducing local inflammation after surgery. In fact, in a small RCT of colorectal cancer patients, the patients who received probiotics preoperatively had maintained normal levels of sIgA after colectomy compared to the control group [75].

- Anastomotic Leak

The potential beneficial effect of probiotics in the prevention of anastomotic leaks is currently being investigated (NCT05164887) [76]. For example, it was shown that intestinal dysbiosis in the form of low diversity and virulence shift of Enterobacteriaceae, Pseudomonas aeruginosa, and Enterococcus faecalis plays an important role in the pathophysiology of anastomotic leaks [25,77].

- Intestinal Motility

Moreover, there is scientific evidence that perioperative administration of probiotics has a beneficial effect on intestinal motility after colorectal surgery. A recent meta-analysis of 21 RCTs involving 1776 patients with gastrointestinal cancer showed that compared with the control group, administration of probiotics and symbiotics either alone or in combination significantly reduced the incidence of abdominal distension (Relative Risk (RR) 0.62) and the incidence of postoperative ileus (RR, 0.47). More specifically, participants receiving probiotics and symbiotics experienced shorter first flatus (Mean Difference (MD), −0.53 days), shorter first bowel movement (MD, −0.78 days), shorter first solid feed (MD, −0.25 days), shorter first liquid feed (MD, −0.29 days), and shorter postoperative hospital stay (MD, −1.43 days) [78].

Data from meta-analysis with large numbers of participants provided evidence that probiotic administration significantly reduces the incidence of antibiotic-associated diarrhea in surgical and nonsurgical patients [32,36,79,80]. This favorable outcome appears to be related to the type, dose, and different combinations of strains administered in each case [79]. Additionally, probiotics could potentially prevent chemoradiotherapy-induced diarrhea [81].

- Postoperative Pain

It is possible that probiotics may help relieve postoperative pain [77]. Rousseaux et al. found that oral administration of *Lactobacillus* spp. based probiotics perioperatively can increase patient pain threshold by interfering with opioid and cannabinoid receptors in intestinal epithelial cells in a similar manner as morphine [82]. In addition to potentially reducing infectious and non-infectious complications, patients who received probiotics also report a better quality of life [36,83,84].

##### Systemic Inflammatory Reaction

Perioperative use of pro-/symbiotics may have a substantial role in attenuating systematic inflammatory responses. Patients tend to experience a faster reduction of inflammatory markers, such as C-reactive protein (CRP) and interleukin-6 (IL-6), and increased levels of natural killer (NK) cells activity when they are taking probiotics perioperatively [67,85]. In fact, patients receiving probiotics before surgery tend to exhibit lower postoperative levels of serum IL-6 and CRP when compared to the control group [75]. After Zhang et al., a more recent RCT by Polakowski et al. showed that the 7-day preoperative use of symbiotics for colorectal patients who are going to undergo colectomy is capable of mitigating the inflammatory reaction after surgery, as it was supported by the reduced postoperative levels of both IL-6 and CRP in the symbiotic group compared to the control group [86]. Similar results with an important reduction of IL-6 levels postoperatively were presented by Kotzampasi et al. In their study, they included colorectal patients who took 3 days before and 14 days after surgery a mixture of probiotics. Except for showing the reduction of IL-6 levels, they tried to give a possible pathophysiological mechanism for this result. So, they found that the postoperative production of the cytokines IL-6 and tumor necrosis factor-α (TNF-α) from patients who received probiotics perioperatively was regulated by the expression of SOCS3. This was supported by the positive correlation between SOCS3 and the levels of IL-6 and the expression of TNF-α [87]. However, in another small RCT where patients with adenocarcinoma underwent resection, they did not find a remarkable difference in postoperative levels of CRP, IL-6, or leukocytes either in patients who received preoperative probiotics or in patients who received symbiotics. With the results being not statistically significant, they only found an increase of IL-6 levels in the symbiotic group in contrast to the control and probiotic group [88]. In accordance with the previous study, other investigators have found no relation between the use of perioperative probiotics/symbiotics and the attenuation of postoperative inflammatory markers [69,70,71,73,89,90]. Furthermore, in a meta-analysis that included studies with obese patients who were scheduled to have bariatric surgery, it was found that the perioperative use of pro-/pre-/symbiotics did not have a statistically significant effect on postoperative levels of CRP, IL-6, and TNF-α [91].

Similar to the results in colorectal patients, CRP and leukocyte levels were significantly decreased postoperatively in biliary cancer patients who received perioperative symbiotics and underwent hepatectomy [59,60]. Interestingly, in one of these two RCTS, Sugawara et al. exhibited a remarkable decrease of postoperative IL-6 levels in the symbiotic group and a boosted NK cell activity when symbiotics were given preoperatively [59]. The same influence of perioperative symbiotics on IL-6 levels was reported in the study of Usami et al. who used symbiotics on liver cancer patients before hepatectomy [72]. However, in a recent RCT where patients with hepatocellular carcinoma received probiotics preoperatively, the IL-6 levels after hepatectomy were not statistically different between the control and the probiotic group [92]. In another RCT with obese patients having a gastric bypass, the perioperative use of probiotics decreased the levels of CRP, IL-6, and TNF-α in the probiotic group after surgery, but this reduction was statistically important only for TNF-α [93].

Another biomarker that could be indicative of the impact of pro-/symbiotics on the inflammation process is the level of immunoglobulins. In fact, in an RCT, patients who took preoperative probiotics had higher serum levels of IgG after surgery, showing that probiotics could play a protective role in the inflammatory process perioperatively [75]. Furthermore, probiotics seem to affect the levels of plasma endotoxin, as it is shown that their perioperative use results in a significant reduction of its serum concentration postoperatively [64]. A small RCT showed that colorectal cancer patients who received preoperative probiotics had significantly lower levels of plasma endotoxin, D-lactic acid, interleukin-6, and C-reactive protein and higher levels of IgG and IgA immunoglobulins after colectomy compared to the control group [75]. On the other hand, Roussel et al., recently, showed that there is no regulatory effect of preoperative probiotics on serum endotoxins as they did not find a statistically important difference between the control group and the liver cancer patients who took probiotics before hepatectomy [92]. In a recent study, the use of probiotics in gastric cancer patients only postoperatively (starting 3–5 days after gastrectomy) showed a notable effect in reducing the inflammatory process provoked by surgery and enhancing existing immunity. This was shown by the important reduction of leukocytes, interleukin-1β (IL-1β), IL-6, and TNF-α and increased levels of immunoglobulin A and immunoglobulin M in the serum [94].

Moreover, probiotics may contribute in a more comprehensive manner to recovery after colorectal surgery by establishing an intestinal microbiota equilibrium and by reducing postoperative inflammation. In an RCT of 60 patients suffering from colorectal cancer in Japan treated with probiotics, namely Bifidobacterium longum BB536, before and after colon resection, the group taking probiotics tended to have lower blood inflammatory markers [85]. The incidence of SSIs was higher in the control group but not statistically significant [85]. However, there was no statistically significant difference in postinfectious complications between the two groups [85]. Additionally, Xu et al. demonstrated that preoperative oral administration of glucose solution combined with postoperative probiotics in patients who underwent colorectal cancer surgery contributes to the reconstitution of gut microbiota, protects the intestinal mucosal barrier function, reduces intestinal mucosal damage and permeability, and improves the systemic inflammatory response [95]. In addition, patients with CD who underwent ileotomy and received probiotics postoperatively had lower inflammatory markers and a lower rate of disease recurrence compared to patients who received placebo, but this result did not reach statistical significance [96].

Last, it is of great interest that in recent years technology has made it possible to genetically modify probiotics to have specific properties that have the potential to prevent and treat disease in humans. Genetically modified probiotics have been used in experimental mouse models. They have been found to influence inflammatory processes in the gastrointestinal tract and to have an inhibitory effect on infections caused by various pathogens such as Pseudomonas, Enterohaemorrhagic *E. coli*, Staphylococcus aureus and *S. epidermidis*, Salmonella, and Vibrios [97]. Moreover, genetically engineered probiotics attenuate inflammation by modulating intestinal microbiota and decreasing ROS in mice with promising implications for inflammation regulation in humans also [98].

In general, probiotics are well-tolerated in the majority of studies [79]. However, there are reports of extremely low incidence of adverse events, such as septicemia, fungemia, and intestinal ischemia, in immunocompromised and critically ill patients. Therefore, caution should be exercised in selecting the appropriate species for specific indications and populations [99,100]. Overall, considering the positive effect of probiotics on reducing postoperative complications, some authors recommend their use in patients undergoing surgical procedures on the digestive system [36,83]. It is true that several systematic reviews have been conducted on the effectiveness of probiotics in surgery; however, the certainty of evidence remains undetermined due to study heterogeneity and conflicting results [32]. More specifically, heterogeneity is evident in terms of study design, strain formulations, type of species, number of microorganisms administered, and timing and duration of therapy [34]. Therefore, large multicenter randomized control trials are needed to determine the ideal treatment regimen, as well as the timing and duration of treatment and to assess outcomes in different populations.

There are some limitations to this narrative review. First, it is possible that our search strategy may have missed some publications of smaller studies. Second, the included studies were significantly heterogeneous as the duration, the dose, the types, the concentration, and the administration route (perioperative, preoperative, postoperative, or combination) of probiotics or/and symbiotics varied importantly among them. In addition, some of the retrieved studies had small sample size, which may have impacted the reliability and validity of their findings.

## 4. Conclusions

In this review, the evidence linking perioperative use of probiotics or symbiotics with modulation of surgery-induced inflammation and the reduction of associated infectious and non-infectious complications is presented. Taking into account all the information described in detail in this review, it could be postulated that probiotics or symbiotics exert their protective effects by reducing local inflammation via (i) increasing SCFAs, which help maintain intestinal permeability and sIgA, which promotes local immune responses; (ii) decreasing zonulin and increasing the lactulose/mannitol ratio, which enhances epithelial cell junctions; and (iii) reducing anastomotic leakage and increasing intestinal motility and by modulating systemic inflammation via (i) reducing IL-6, CRP, and TNF-α, in a SOCS3 dependent manner, endotoxin decrease, or through a different undetermined pathophysiologic pathway; (ii) enhancing NK cell activity; and (iii) increasing serum levels of immunoglobulins. Many studies and meta-analyses have been conducted to examine the efficacy of probiotics and symbiotics perioperatively in reducing post-surgical inflammatory complications with promising, albeit often conflicting results. However, the exact pathophysiologic alterations and impact of those preparations on local and systemic inflammatory responses remain undetermined. Gut microbiota are responsible for immunity, growth, and a multitude of metabolic functions through different mechanisms that can indirectly affect both its bacterial composition and its role in the human organism. Restoring gut microbiota after disruption caused by surgery, stress, or antimicrobial use may accelerate local healing processes, attenuate systemic inflammation, and prove beneficial to certain populations. More research is currently needed in order to yield strong evidence for formulating targeted interventions and recommendations.

## Data Availability

No new data were created or analyzed in this study. Data sharing is not applicable to this article.
